# A combination of COVID-19 and dengue fever in Bangladesh: Preparedness of Bangladesh

**DOI:** 10.7189/jogh.10.020314

**Published:** 2020-12

**Authors:** Promit Barua Chowdhury, Sorif Hossain, Raaj Kishore Biswas

**Affiliations:** 1 Institute of Statistical Research and Training, University of Dhaka, Bangladesh; 2 Transport and Road Safety (TARS) Research Centre, School of Aviation, University of New South Wales, Australia

The COVID-19, the novel coronavirus, was first identified in Wuhan city, Hubei Province, China on 31 December 2019. The World Health Organization declared the COVID-19 a global pandemic on 11 March 2020, first since H1N1 influenza in 2009 [1]. In Bangladesh, the first case of COVID-19 was found on 7 March. The highest single day death toll (N = 53) and the highest single day infection (N = 3862) was recorded on June 16. The total number of recorded deaths due to COVID-19 was 1262 as of June 16 from total 533 717 test with 94 481 positive cases [2]. Bangladesh underwent a countrywide lockdown from 26 March, which was periodically extended up to 30 May, following a surge in infections on 13 May and since then the most vulnerable areas are under experimental lockdown. Restrictions are strictly imposed on transportation except for emergency vehicles. A fund of 12.5 million BDT is allocated for the poor and badly affected due to the outbreak [3].

A shortage of testing kits and other medical facilities were observed in Bangladesh since the early onset of the COVID-19 outbreak, despite continuous insistence on ‘well-prepared health system’ by the authorities [4]. As a result, only 11 223 tests were conducted on the first 5 weeks, which was the lowest worldwide testing rate [2]. Even the treatments for COVID-19 were delayed due to inadequate protective equipment in the health facilities. As a result, high infection rate among health workers were observed along with 35 reported cases of deaths of doctors [5]. Many health care workers refused to provide services, which lead to an increased fear and anxiety in the community. However, at the end of April the number of resources were increased, with currently 60 test centers alongside a stock of 1 392 601 personal protect equipment, 13 284 isolation beds, 3 135 420 masks, 562 439 gloves, and 179 759 hand sanitizers [6].

Besides the ongoing pandemic, Bangladesh faced the onslaught of dengue last year (2019) with 2020 season expected to start in June, a viral infection caused by four types of viruses (DENV-1, DENV-2, DENV-3, DENV-4) from the Flaviviridae family. These viruses are typically transmitted infected *Aedes aegypti* and *Aedes albopictus* female mosquitoes. These mosquitoes thrive in areas withstanding water, including puddles, water tanks, containers, and old tires. The lack of reliable sanitation and regular garbage collection also contribute to the spread of the mosquitoes. Dengue infection could be asymptomatic, which makes it highly transmissible. Generally, the infection is characterized by flu-like symptoms including multiple waves of sudden high fevers, which may progress to the Dengue Hemorrhagic Fever (DHF) [7]. Similar to COVID-19, symptomatic treatments are the only cure for the DHF. Despite being an irregular event in the past, Bangladesh experienced its deadliest outbreak of dengue fever with 101 354 reported cases and 179 deaths in 2019 [8].

**Figure 1:**
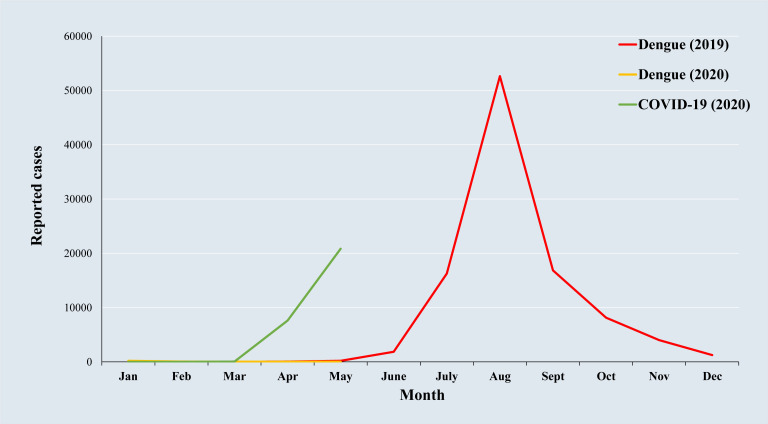
Dengue cases for 2019 and 2020, and COVID-19 cases up to May 21, 2020.

The combination of dengue fever and COVID-19 (**Figure 1**) would aggravate the already stretched health system of Bangladesh. A multicenter early needs assessment study warned that dengue and natural disasters could worsen the COVID-19 crisis in Bangladesh [9]. The assessment study also identified key indicators of risks exposures to COVID-19 including congested urban focused unsustainable vulnerability, demographic and social vulnerability, economic and physical vulnerability, and recurrent disaster vulnerability, which listed the 20 most vulnerable districts out of total 64. The efforts of health sector are now concentrated on controlling the COVID-19 pandemic, which also needs to prepare for dengue outbreak immediately before any further escalation.

**Figure:**
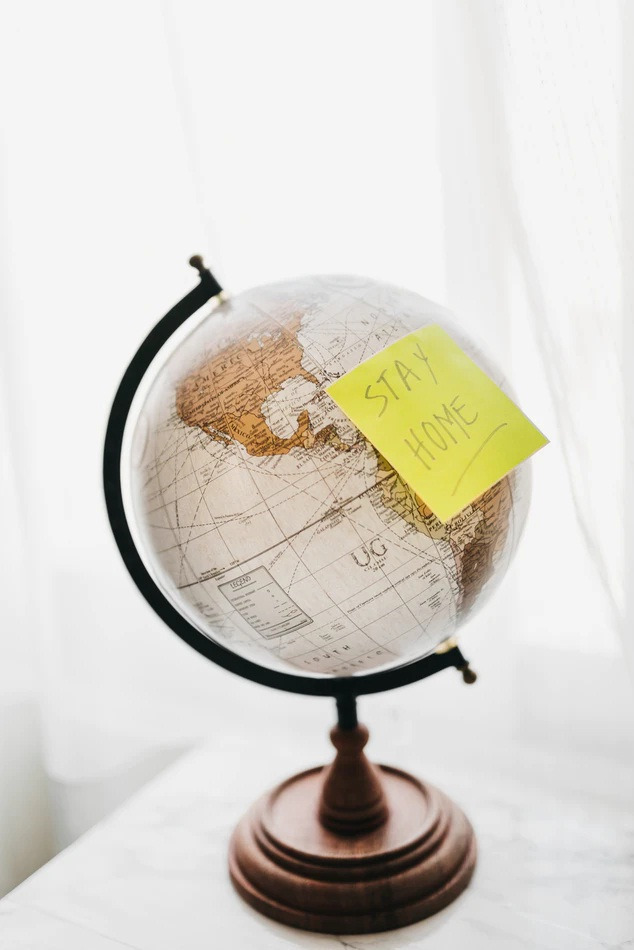
Photo: From https://unsplash.com/photos/W3IXtchd1pE.

With the onset of the monsoon season expected between the end of May to September, the density of Aedes population is predicted to rise. The construction sites are considered as the main sources of Aedes breeding as well as the currently idle terminal stations. If the dengue epidemic spreads at a similar rate of the COVID-19, it would be impossible to equip health facilities to treat patients suffering from both the COVID-19 and the DHF. In overcrowded health facilities, maintaining social distance would be challenging. Although the number of tests has increased over the last few weeks, the rate of test conducted is still 3242 tests per million (as of 16 June 2020), which is insufficient for a country with around 161.4 million population. The scenario would get worse due to the similar nature of symptoms for these two diseases. The delay of identifying the sources of flu-like symptoms, be it from dengue fever or coronavirus, would affect treatment speed as well as recovery rate.

The Local Government Rural Development (LGRD) department has allocated 3 billion BDT (US$35 million) for cleaning and joint awareness campaigns for COVID-19 and dengue fever [10]. However, data deficiency has created multifaceted problems for the policymakers [4]. Masks, sanitizers, gloves, and PPEs (for front line workers) should be in manufactured widely and be made a part of daily lives. Evidence based policies need to in place for lockdown relaxation, which could consider slowly opening districts with low infection rates and continuous random sampling to monitor progress [11]. The rules of social distancing in business or industrial zones should be strictly maintained as well as surveilling environments that are breeding grounds for Aedes. Bangladesh need to be better prepared for dengue than it was for the COVID-19, otherwise a disaster awaits for the health sector.
